# Daily yogurt consumption does not affect bone turnover markers in men and postmenopausal women of Caribbean Latino descent: a randomized controlled trial

**DOI:** 10.1186/s40795-023-00800-2

**Published:** 2024-01-11

**Authors:** Lindsay McGrail, Daniela Vargas-Robles, Mayra Rojas Correa, Lisa C. Merrill, Sabrina E. Noel, Martha Velez, Ana Maldonado-Contreras, Kelsey M. Mangano

**Affiliations:** 1 Department of Biomedical and Nutritional Sciences, University of Massachusetts, 3 Solomont Way, Lowell, MA 01832 USA; 2 Center for Population Health, UMass Movement Research Center, University of Massachusetts, Lowell, MA USA; 3 Department of Microbiology and Physiological Systems, Program of Microbiome Dynamics, University of Massachusetts Chan Medical School, Worcester, MA USA; 4 Department of Health and Human Services, City of Lawrence, Lawrence, MA USA

**Keywords:** Yogurt, Bone turnover markers, Gut microbiota, Inflammation, Aging

## Abstract

**Background:**

Caribbean Latino adults are at high risk for osteoporosis yet remain underrepresented in bone research. This increased risk is attributed to genetics, diet, and lifestyle known to drive inflammation and microbial dysbiosis.

**Objective:**

The primary objective of this study was to determine whether consuming 5 oz of yogurt daily for 8wks improves bone turnover markers (BTMs) among Caribbean Latino adults > 50 years; and secondarily to determine the impact on the gut microbiota and markers of intestinal integrity and inflammation.

**Methods:**

Following a 4wk baseline period, participants were randomized to an 8wk whole fat yogurt intervention (*n* = 10) daily, containing only *Streptococcus thermophilus* and *Lactobacillus bulgaricus,* or to an untreated control group that did not consume yogurt (*n* = 10). Blood and stool samples collected at week-0 and week-8 were used to assess BTMs, inflammation, intestinal integrity biomarkers, and gut microbiota composition, short chain fatty acids (SCFAs), respectively. Data were evaluated for normality and statistical analyses were performed.

**Results:**

Participants were 55% women, with a mean age of 70 ± 9 years, BMI 30 ± 6 kg/m^2^, and serum C-reactive protein 4.8 ± 3.6 mg/L, indicating chronic low-grade inflammation. Following 8wks of yogurt intake, absolute change in BTMs did not differ significantly between groups (*P* = 0.06–0.78). Secondarily, absolute change in markers of inflammation, intestinal integrity, and fecal SCFAs did not differ significantly between groups (*P* range 0.13–1.00). Yogurt intake for 8wks was significantly associated with microbial compositional changes of rare taxa (*P* = 0.048); however, no significant alpha diversity changes were observed.

**Conclusions:**

In this study, daily yogurt did not improve BTMs, inflammation, intestinal integrity, nor SCFAs. However, yogurt did influence beta diversity, or the abundance of rare taxa within the gut microbiota of the yogurt group, compared to controls. Additional research to identify dietary approaches to reduce osteoporosis risk among Caribbean Latino adults is needed.

**Trial registration:**

This study is registered to ClinicalTrials.gov, NCT05350579 (28/04/2022).

**Supplementary Information:**

The online version contains supplementary material available at 10.1186/s40795-023-00800-2.

## Background

Deterioration of bone structure and strength is a consequence of osteoporosis (OP), a disease in which bone remodeling becomes imbalanced, favoring bone resorption and leading to loss of bone mineral density (BMD) and weakening of microarchitecture. Development of OP is strongly associated with natural aging and physiological decline, however other comorbidities may contribute to disease exacerbation, including intestinal dysfunction [1–5]. A bone fracture due to OP is common, which may lead to long-term disability, institutionalization, and decreased quality of life [4]. Medications to treat OP have poor compliance due to negative side effects, supporting the need to establish effective, and sustainable strategies to mitigate the risk and progression of OP [6].

Bone remodeling is a tightly regulated process where resorption and formation occur in tandem to renew old and damaged bone, contributing to skeletal integrity and homeostasis. The dynamics or rate of bone remodeling can be indirectly evaluated in blood by bone turnover markers (BTMs), such as tartrate-resistant acid phosphatase subunit 5b (TRAP5b), an enzyme produced by osteoclasts during resorption, and pro-collagen I alpha 1 (P1NP) and osteocalcin (OC), residual proteins released during bone formation [7]. When the remodeling process favors resorption over formation, bone homeostasis is disturbed and can lead to OP [8]. Historically, OP has been considered a disease that primarily affects non-Hispanic White women. However, recent work from the Boston Puerto Rican and National Health and Nutrition Examination cohorts shows age-adjusted prevalence of low bone mass and OP is greater among Puerto Rican men compared to rates in other ethnic groups, and rates among Puerto Rican women are similar to the high rates observed in non-Hispanic White women [9]. Individuals of Caribbean Latino or Hispanic descent (henceforth referred to as Caribbean Latino) are underrepresented in nutrition and bone-related research. Despite these estimates, this population will have one of the largest increases in fracture incidence and incur more than a doubling of health care costs related to osteoporotic fractures by 2025 [10]. Culturally acceptable lifestyle modifications to improve bone health among Caribbean Latino adults are urgently needed.

Diet is a modifiable factor that can elicit protection against OP through the maintenance of BMD and reduced fracture risk [11, 12]. Specifically, the consumption of yogurt may protect bone through the provision of bone beneficial nutrients including calcium, vitamin D (only when fortified), and protein [13, 14]. Studies from a similar Caribbean Latino population relocated to the Boston area from the Island of Puerto Rico showed calcium intakes below the recommended guidelines [15, 16]; a potential contributor to the reported high risk of OP in this population. Yogurt provides calcium and probiotics, and has been found to reduce inflammation, improve intestinal integrity, and modulate the gut microbiota composition and metabolic function [13, 17–20]. Growing evidence suggests that gut microbial homeostasis elicits bone protective attributes through an abundance of health-promoting bacteria, and functional anti-inflammatory metabolites, including short-chain fatty acids (SCFA) [21–23]. In contrast, dysbiosis or the reduction of beneficial bacteria in favor of putrefactive ones, has been found to deteriorate intestinal health through increased inflammation and loss of intestinal integrity [24], contributing to bone turnover dysregulation [25]. Yogurt may impact bone by reducing microbial dysbiosis; however, yogurt intake among Caribbean Latino adults is low, with consumption at approximately 1 serving per week [16]. This randomized controlled trial aimed to determine the effect of 8-weeks of daily yogurt intake on blood BTMs among Caribbean Latino adults at risk for OP, who minimally consume yogurt, and have chronic inflammation at baseline. A secondary objective was to identify mechanisms underpinning yogurt’s effect on bone health, including the gut microbiota, SCFA metabolism, inflammation, and intestinal integrity.

## Methods

### Participants

Caribbean Latino men and women, over the age of 50 were recruited through advertisements and announcements at the Center (a recreation and service center primarily serving older adults) in Lawrence, MA, between February 2018 and September 2018 (study recruitment was paused for 8 months due to the Merrimack Valley gas explosions, displacing thousands of people from their homes) and enrollment from April 2019 to February 2020 (study enrollment and completion was terminated early due to the COVID-19 pandemic in March 2020). Interested individuals were interviewed in either English or Spanish, by a trained bilingual interviewer, to verify eligibility. Study inclusion criteria included at least 50 years of age and self-reported Caribbean Latino descent. Exclusion criteria included yogurt intake > 2 servings per week, antibiotic use in the past 6 months, regular use of laxatives, self-reported cancer, gastrointestinal diseases, gastrointestinal alteration procedure (appendectomy, gastric bypass surgery), osteoporosis, osteoporosis-related fracture, and self-reported current use of osteoporosis drugs, steroids, or chemotherapeutic agents. The University of Massachusetts Lowell Institutional Review Board approved all study protocols (No. 18–095-MAN-XPD). 

### Study design and participant timeline

**Fig. 1:**
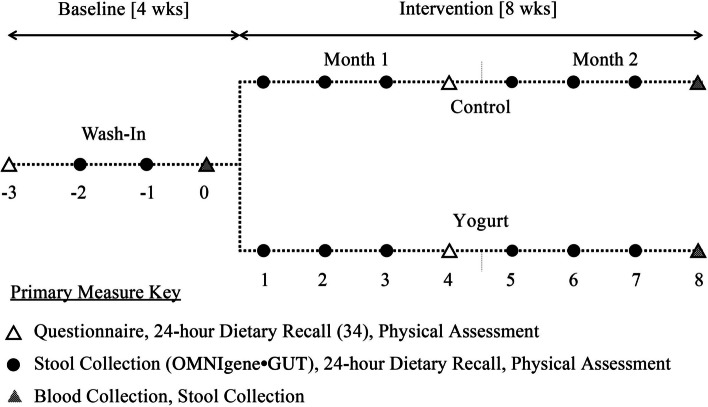
Timeline of randomized controlled trial conducted among older Caribbean Latino men and women assigned to either a non-yogurt diet-control or yogurt intervention group

On a rolling admission basis, an independent research manager randomly assigned participants to the yogurt or non-yogurt diet-control group (referred to as ‘control’ group who did not receive treatment), stratified by sex in Excel. Participants, who were 55% women, with a mean age of 70 ± 9 years, had a BMI of 30 ± 6 kg/m^2^, and chronic low-grade inflammation defined by serum C-reactive protein at 4.8 ± 3.6 mg/L, attended 12 weekly visits with study staff, all of whom where unblinded (Fig. 1).

**Fig. 2:**
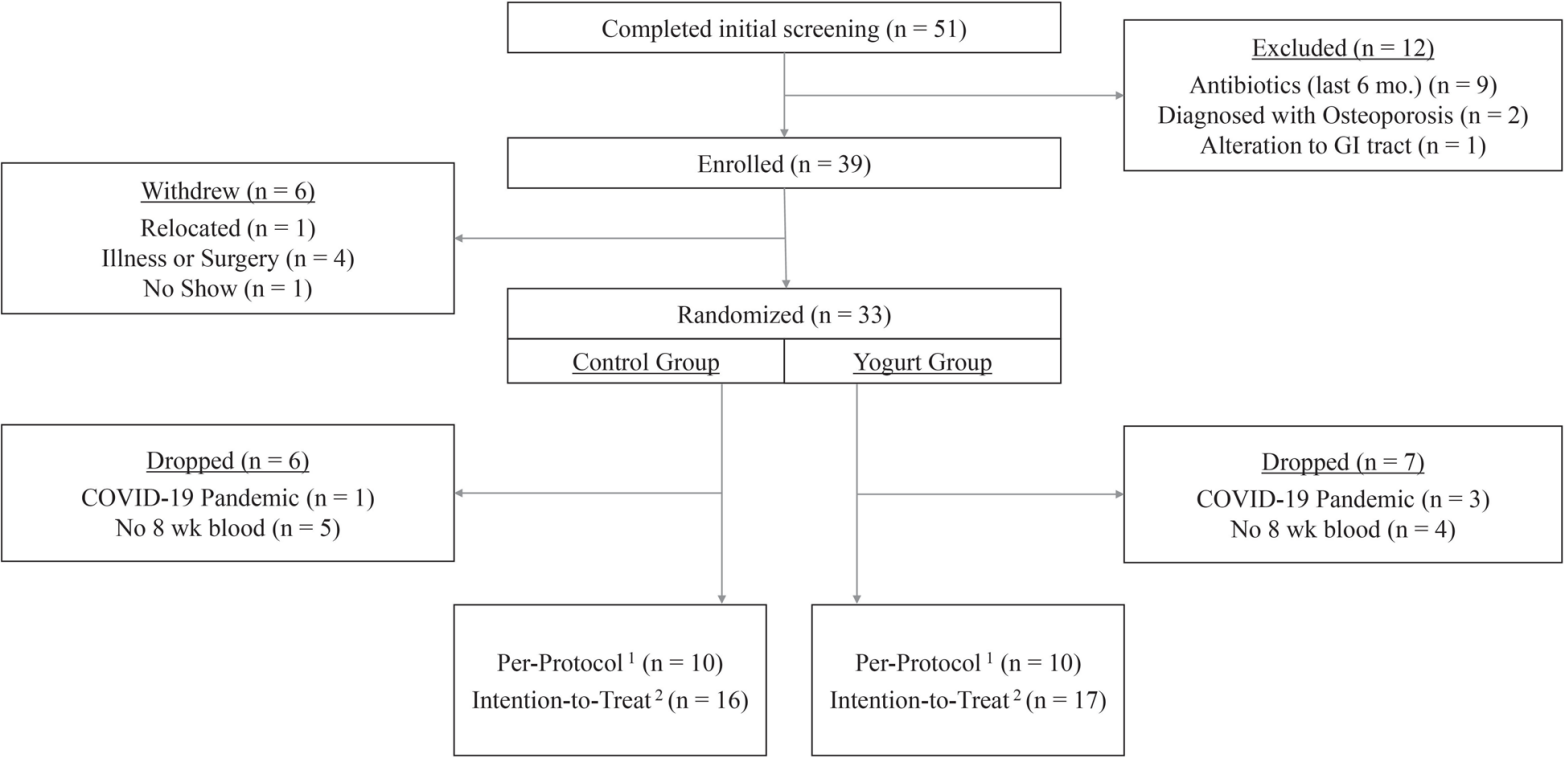
Consort diagram of the randomized controlled trial conducted among older Caribbean Latino men and women assigned to either a non-yogurt diet-control or yogurt intervention group. ^1^Per-protocol analyses exclude all participants without a blood sample collected at 8-weeks ± 7 days. ^2^Intention-to-treat analyses used the last observation carried forward method for all participants with a missing sample at 8-weeks ± 7 days

To assess baseline health, diet, BTMs, gut microbiota, SCFAs, inflammatory, and intestinal integrity biomarkers, participants partook in a 4-week baseline “wash-in” period (weeks -3, -2, -1, 0), followed by the 8-week intervention period. All participants were encouraged to maintain their normal diet throughout the study period. The control group was asked to abstain from yogurt consumption. Diet was monitored by weekly 24-h dietary recalls, which helped to verify yogurt intake among both groups. Due to the study visits revolving around the timing of stool production and participant scheduling needs, dietary assessment during weekdays and weekend varied within and across participants. Figure 2 illustrates participant enrollment and retention. Following study completion, control group participants were offered 8 weeks of daily yogurt, free of cost, and all individuals received a $125 supermarket gift card for their commitment and participation.

### Intervention

Yogurt assigned participants were provided with a biweekly supply, were directed to consume one 141-g (5-oz) yogurt daily and were asked to report time of consumption and any changes to bowel health. Supplemental Table 1 shows the nutrient content of each serving of yogurt. In brief, 100 g of the yogurt provided 121 kcal, 5.7 g total fat, 11.3 g total carbohydrate, 3.5 g of protein and 10.6% of daily calcium. The ingredients include Pasteurized Grade A Milk, Cane Sugar, Yogurt Cultures (*L. bulgaricus*, *S. thermophilus*), and Vanilla Extract. Due to the typical non-consumption of yogurt in this population, a small group of Caribbean Latino adults at the Center participated in a survey and reported preferences for yogurt texture and flavor prior to study initiation. The intervention chosen for this study was based on the survey results. Additionally, the yogurt had the desired nutrient profile, met the Codex Alimentarius definition of yogurt by containing *S. thermophilus* and *L. bulgaricus* [26], and lacked preservatives, added fruit or pectin.

### Demographics, health & dietary patterns

Medical history was collected and managed using REDCap electronic data capture tools [27]. At baseline, participants self-reported sex, age, education, household income, marital status, employment status, place of birth, migration history, level of acculturation [28–30], alcohol consumption defined as heavy (> 1 (women), > 2 (men) per day), moderate (1 (women), 2 (men) per day), or never, and physical activity level used to calculate a physical activity score in metabolic equivalents (METs) per week [31, 32]. Additionally, self-reported health and behaviors including medical diagnoses, prescription use, over-the-counter medication use, bowel patterns according to the Bristol Stool Scale [33], and smoking status (current/former/never) were collected through an interviewer administered verbal questionnaire (supplementary file) at baseline. Changes to baseline health and behaviors were monitored through interviewer administered questionnaires at week -3, 1, 4, and 8 of the study. Baseline and follow-up questionnaires were developed for The Boston Puerto Rican Health Study [34] but were modified for bowel pattern information and tracking. A physical assessment at each visit was performed, including duplicate measures of body weight in kilograms (kg) and height in meters (m) to calculate BMI (kg/m^2^). Participants completed an interviewer administered 24-h dietary recall at weekly visits to obtain detailed information on food and beverage consumption. Dietary recalls were collected using the USDA Automated Multiple Pass Method [35] in conjunction with the University of Minnesota Nutrition Data System for Research software (NDSR 2018, NCC, University of Minnesota, Minneapolis). Participants were prompted with food models to aid portion size estimation. Four 24-h dietary recalls were collected per month and mean ± SD was calculated for calories, protein, fat, carbohydrates, dairy (sum of milk, cheese, or fermented dairy products both including and excluding the intervention), as well as dietary fiber, vitamin D, and calcium. All interviewers were bilingual to alleviate language barriers for study participation.

### Stool sample collection

Participants were provided detailed written and video instructions, at an eighth-grade reading level in both English and Spanish, for at-home, self-collection of stool samples into OMNIgene•GUT kits (Genotek, Canada). Participants were also provided printed instructions, gloves, a stool collection hat, an OMNIgene•GUT kit, and brown storage bags [36]. After sample production, participants were instructed to add the sample to the tube, close the tube, and invert it for 30 s to resuspend the sample into the DNA stabilization solution, then refrigerate it at home until provided at the next visit. Samples were received at the Center within 2–5 days of production where they were refrigerated. Upon arrival at the laboratory, samples were aliquoted into cryovials and stored at -80˚C. Total DNA was isolated from one of each sample using the MagAttract PowerSoil DNA EP Kit (QIAGEN, Inc.).

### Blood sample collection

At week 0 and week 8, trained phlebotomists collected three vacutainers of non-fasted blood from all the participants at the Center. Samples were transported to the laboratory using standard protocol within 3.5 h of draw. A complete blood count was performed using a Horiba ABX Micros ES60 instrument; these results were used to evaluate underlying health status, normal function, and infection confounders. Serum and plasma were separated via centrifugation at 4500 × g at 4ºC for 15 min, then were aliquoted into cryovials and stored at -80ºC until analyses.

### Intervention compliance

To evaluate compliance to the intervention or diet-maintenance, the relative abundance of the bacterial cultures provided by the intervention yogurt (*S. thermophilus* (ST), *L. bulgaricus* (LB)) within the participants’ gut was assessed via quantitative real-time polymerase chain reaction (qPCR), using DNA extracted from three stool samples. Primers, validated by Stachelska et al. [37], were designed to target a region of the *lacZ* gene that codes for beta-galactosidase production. For normalization of total bacteria, *16S rRNA* (16S) was used as an endogenous control. Primers were ordered through Integrated DNA Technologies, as presented in Supplemental Table 2. The Bio-Rad master mix, iTaq Universal SYBR Green Supermix (#1,725,120, Hercules, CA) was used, and thermal cycling parameters were set according to the manufacturer’s protocol. The delta-delta Ct algorithm (2 ^–(∆∆Ct)^) was applied to threshold values obtained from the thermal cycler to evaluate relative abundance of yogurt starter cultures in stool.

### Bone turnover marker assessment

Markers of bone turnover were quantified in serum via enzyme-linked immunosorbent assay (ELISA). Quidel Corporation ELISA kits were used to quantify TRAP5b (8033), and Abcam ELISA kits were used to quantify P1NP (ab210966) and OC (ab270202), according to the manufacturer’s protocols. Sera collected at week 0 and week 8 were diluted to achieve concentrations within the assay range and sample absorbance at 405 nm (TRAP5b) or 450 nm (P1NP, OC) was measured using a Tecan Infinite M200 Pro microplate reader paired with Tecan Magellan Software (version 7.0). All raw data were blank reduced, adjusted for dilution, and interpolated using the standard curve.

### Gut microbiota profiling

To evaluate bacterial phylogeny and taxonomy, stool DNA was amplified at the V4 region of bacterial *16S rRNA*gene using universal primers 515F/806R, as previously described [36]. Libraries were sequenced using the Illumina MiSeq platform, following a 2 × 300 base pair paired-end protocol. Quality-filtered and demultiplexed reads were processed as previously described [36, 38]. Forward and reverse *16S*MiSeq-generated amplicon sequencing reads were dereplicated and sequences inferred using DADA2 [39]. Potential chimeric sequences were removed using consensus-based methods. Taxonomic assignments were made using BLAST against the NCBI RefSeq RNA database. The rooted phylogenetic tree was built in QIIME 2 2021.4 [40]. First, amplicon sequence variant (ASV) sequences were aligned with mafft [41] (via q2‐alignment) and then phylogeny was constructed with fasttree [42] (via q2‐ phylogeny align-to-tree-mafft-fasttree). The table of ASV abundances was rarified at 5,612 sequences per sample, from which no samples were lost. The mean total was 271 ± 59 ASVs per sample (see Supplemental Table 3).

### Short-Chain fatty acid profiling

Ten SCFA isomers (acetate, propionate, butyrate, isobutyrate, valerate, isovalerate, 2-methylbutyrate, 3-methylvalerate, 4-methylvalerate, hexanoate) were quantified via liquid chromatography-tandem mass spectrometry, following an adapted, validated protocol [43]. Due to the low molecular weight of SCFAs, all samples and standards were converted to their 3-nitrophenylhydrazones for improved precision and accuracy. Additionally, a mixed SCFA stock was isotopically labeled through derivatization, which was used as an internal standard (IS) calibrator for each sample. Derivatizing reagents, 200 mM 3-nitrophenylhydrazine (3-NPH) and 120 mM 1-(3-dimethylaminopropyl)-3-ethylcarbodiimide hydrochloride (EDC)-6% pyridine, were prepared in 50% aqueous acetonitrile. The SCFA stock was equally mixed with EDC-pyridine/3-NPH for preparation of the standard curve, or EDC-pyridine/^13^C_6_-3-NPH for the isotope-labeled IS. All derivatization synthesis occurred at 40ºC for 30 min on a heating block. Lyophilized stool samples were weighed then resuspended with 50% acetonitrile, vortexed, then centrifuged at 2000 × g for 5 min. The supernatant (stool SCFA extract) was diluted 1:100, mixed, and combined equally with 3-NPH and EDC-pyridine for derivatization. Each derivatized standard or sample was equally mixed with the IS in a vial and placed into the autosampler for analysis using an injection volume of 10 μL. Separation of all isomers was achieved using a Kinetex F5 (2.6 μm 100 Å, 100 × 4.6 mm).

### Inflammatory & intestinal integrity biomarker assessment

Eight inflammatory cytokines (TNF-α, IL-6, IL-10, IFN-γ, IL-1β, IL-8, IL-12p70, MCP-1) were quantified using the FirePlex Human Inflammation—Immunoassay Panel (ab243550, Abcam, Cambridge, MA). Raw data were blank reduced, adjusted for dilution, and standard curve interpolated. A pro-inflammatory cytokine score was calculated for each participant by ranking the inflammatory cytokines by tertile. For all cytokines, except IL-10, the top tertile of data (≥ 67%) received a rank of 2, the middle tertile data (33—66%) received a rank of 1, and the bottom tertile of data (< 33%) received a rank of 0. Due to the anti-inflammatory properties of IL-10, the ranking system was opposite, where the top tertile of IL-10 values received a rank of 0. The rankings were summed to produce a maximum score of 16, indicating highest inflammation, and a minimum score of 0, indicating lowest inflammation. To independently assess chronic low-grade inflammation, CRP was quantified in serum using the automated Medica EasyRA Clinical Chemistry Analyzer. Chronic low-grade inflammation is defined by CRP values greater than 3 mg/L but less than 10 mg/L [44]. Intestinal integrity biomarkers, lipopolysaccharide binding protein (LBP) and intestinal fatty acid binding protein (FABP2) were measured in plasma using Abcam ELISA kits (ab213805, ab234566). Sample absorbance at 450 nm was measured using Tecan Infinite M200 Pro microplate reader paired with Tecan Magellan Software. All raw data were blank reduced, adjusted for dilution, and interpolated using the standard curve.

### Statistical analyses

Statistical tests were conducted using the SAS 9.4 software (SAS Institute Inc., Cary, NC), with a significance level of α = 0.05. Per-protocol and intention-to-treat analyses were performed. Missing datapoints for intention-to-treat analyses were imputed using the last observation carried forward method. Results from the per-protocol and intention-to-treat analyses were similar; therefore, only per-protocol data are presented. Data were evaluated for normality using the Shapiro–Wilk statistic via the univariate procedure. Normally distributed outcomes were compared using t-tests and non-normally distributed variables were log transformed prior to t-test (CRP absolute change, fiber month 0, protein month 2).

Analyses of the gut microbiota were conducted using R Statistical Software [45] (Studio Package v1.4.1106). Alpha and beta diversities, as well as taxa associations were evaluated between groups (yogurt vs. control group) and within groups (baseline vs. intervention) using regression models. Covariates included in the models were age, sex, BMI, use of gut microbiota altering medications (yes/no), grams of fiber per day, kcal per day. To account for repeated measures, the variable “subject” was used as a random variable for all analyses. Alpha diversity analyses we performed fitting linear mixed models (LMM) with “lme” function from the “nlme” [46] package. Alpha diversity was assessed by Shannon index using the “vegan” [47] package. Beta diversity analyses were performed using non-parametric PERMANOVA [48] with adonis2 function from the “vegan” [47] package and using the setblock function from the “permute” [49] package in R to restrict permutations within the repeated measures of each individual. Permutation was set to 1000 and seed was set to 711. Bray Curtis dissimilarities and weighted and unweighted UniFrac distances were utilized among samples. Beta diversity visualization was performed with Principal Coordinates Analysis (PCoA) as an ordination method. Alpha and beta analyses were performed at ASV level. The analyses described above were performed using the “phyloseq” [50] package. Specific microbial taxa associations with yogurt consumption were analyzed using the MaAsLin2 [51] package default parameters, which also allows setting a random variable. MaAsLin2 analyses was performed at species level. To analyze associations of changes in biomarkers and changes in taxa abundance, the table was not normalized or log-transformed since negative values were present. Associations between the absolute change in BTMs, inflammatory, and intestinal integrity biomarkers were compared with change in relative abundance for each taxon from baseline to the last intervention period (week 6.1 to 9). Baseline values were calculated considering the mean abundance of each subject at baseline as the reference and the mean during the final intervention weeks (6.1 to 9). For MaAsLin results, multiple comparisons were adjusted using the Benjamini-Hochberg's false discovery rate (FDR) [52] method. To avoid collinearity among biomarkers, Spearman correlation tests were run to separate variables in different models. Plots were generated using the “ggplot2” [53] package and base R functions or were the output of MaAsLin2 algorithm and edited in Adobe Illustrator.

Based on absolute difference in OC between two dietary supplemented groups at 12 months (dried apples 17.2 ± 0.7 ng/mL versus dried plums 15.6 ± 0.6 ng/mL) [54] a sample size of 20 (10 per group) was calculated with a power of > 0.99 using G*power [55]. In addition, from the same study [54], CRP measured at 3 months compared between supplemented groups (dried apples 20.5 ± 3.0 mg/L versus dried plums 14.9 ± 3.0 mg/L) provided a > 0.99 power with a sample size of 16 per group. To account for potential drop out, the study aimed to recruit 20 participants per group.

## Results

### Demographics, health & dietary patterns

A total of 51 Caribbean Latino men and women, older than 50 years, completed the initial screening, 39 of whom were eligible, provided written informed consent, and enrolled in the study, as shown in Fig. 2. Following enrollment, 6 participants withdrew from the study due to relocation, medical reasons, or unknown reason. A total of 33 participants were successfully randomized, however only 20 participants (*n* = 10/group) provided blood at both week 0 and 8. This loss is due to the COVID-19 pandemic (*n* = 4) or collection outside of the study period of 8 weeks ± 7 days (*n* = 9). Enrollment took place between February 2018 and September 2018 (study recruitment was paused for 8 months due to the Merrimack Valley gas explosions, displacing thousands of people from their homes) and recontinued from April 2019 to February 2020. Study enrollment was terminated early due to the COVID-19 pandemic in March 2020. Characteristics of participants included in the per-protocol analyses are presented in Table 1 by group.

Compared to baseline, there were no changes in weight or BMI in either group following the intervention, and minimal changes in diet were observed, as presented in Supplemental Table 4.

**Table 1:** Baseline characteristics among participants assigned to non-yogurt diet-control (C) or yogurt intervention (Y) group

Participant Characteristics	C (*n* = 10)	Y (*n* = 10)
Women [% (*n*)]	50 (5)	60 (6)
Postmenopausal [%]	100	100
Age (years)	69 ± 8	71 ± 10
Body mass index (kg/m^2^)	31 ± 6	29 ± 5
Percent Acculturated (%)	22 ± 2	42 ± 25
Physical Activity Score (METs/w)	11 ± 5	14 ± 5
Household Income (USD/y)	15,841 ± 8,892	46,462 ± 58,649
Education > 9^th^ grade [% (*n*)]	80 (8)	100 (10)
Consumes Alcohol [% (*n*)]	40 (4)	30 (3)
Smokes Tobacco [% (*n*)]	0 (0)	10 (1)
Anti-inflammatory Medication Use [% (*n*)]	20 (2)	20 (2)
^a^Microbiota Altering Medication Use [% (*n*)]	0 (0)	20 (2)

Data are presented as means ± SD or % (*n*)

^a^Metformin, Omeprazole, proton pump inhibitors, docusate, laxatives

### Intervention compliance

**Fig. 3:**
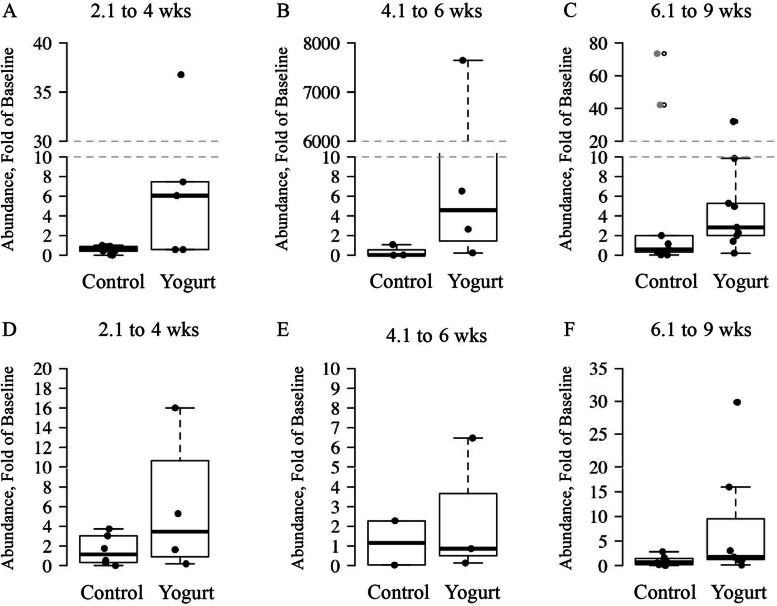
Relative abundance of *S. thermophilus* and *L. bulgaricus* in stool as a measure of intervention compliance conducted among older Caribbean Latino men and women assigned to either a non-yogurt diet-control or yogurt intervention group

The mean abundances of *S. thermophilus* and *L. bulgaricus* at baseline were used as a reference to determine fold-change throughout the intervention and are illustrated in Fig. 3.

Mean relative abundance is expressed as fold-change from baseline ± SD. Due to sample availability, not all participants had data at each timepoint. (A) Relative abundance of *S. thermophilus* in samples collected between 2.1 and 4 weeks into the intervention (C: *n* = 9 | Y: *n* = 5). (B) Relative abundance of *S. thermophilus* in samples collected between 4.1 and 6 weeks (C: *n* = 3 | Y: *n* = 4). (C) Relative abundance of *S. thermophilus* in samples collected between 6.1 and 9 weeks (C: *n* = 9 | Y: *n* = 9). (D) Relative abundance of *L. bulgaricus* in samples collected between 2.1 and 4 weeks (C: *n* = 6 | Y: *n* = 4). (E) Relative abundance of *L. bulgaricus* in samples collected between 4.1 and 6 weeks (C: *n* = 2 | Y: *n* = 3). (F) Relative abundance of *L. bulgaricus* in samples collected between 6.1 and 9 weeks (C: *n* = 8 | Y: *n* = 7).

### Bone turnover marker assessment

Concentrations of resorption marker TRAP5b and bone formation markers P1NP and OC at week 0 and week 8 did not significantly change and are presented in Table 2.

**Table 2:** Baseline and 8-week serum and stool biomarkers among non-yogurt diet-control (C) and yogurt intervention (Y) group

	**Week 0 Collection**		**Week 8 Collection**		
**Biomarkers**	**C (*n* = 10)**	**Y (*n* = 10)**	**C (*n* = 10)**	**Y (*n* = 10)**	***P ***^**a**^
TRAP5b (U/L)	3.1 ± 1.2	3.9 ± 1.6	3.4 ± 1.8	3.6 ± 1.4	0.11
P1NP (ng/mL)	203 ± 93.9	180 ± 75.7	196 ± 109	202 ± 71.4	0.06
OC (ng/mL)	52.2 ± 18.9	43.8 ± 26.3	55.4 ± 31.5	48.7 ± 26.0	0.78
Stool Short-Chain Fatty Acids (µmol/g)					
Acetate	283 ± 54.3	258 ± 77.1	367 ± 114	284 ± 125	0.23
Propionate	46.0 ± 7.8	40.8 ± 8.7	59.9 ± 20.8	43.1 ± 16.5	0.18
Butyrate	20.7 ± 14.4	13.1 ± 10.5	22.5 ± 11.8	13.0 ± 6.4	0.37
Isobutyrate	3.1 ± 1.6	2.2 ± 1.1	2.9 ± 1.7	2.4 ± 0.9	1.00
Valerate	4.2 ± 1.2	3.8 ± 1.9	4.4 ± 2.3	3.7 ± 0.9	0.57
Isovalerate	2.7 ± 1.6	2.3 ± 1.0	2.6 ± 1.6	2.4 ± 1.2	0.97
2-Methylbutyrate	1.8 ± 1.4	1.2 ± 0.9	1.3 ± 0.9	1.2 ± 0.7	0.78
3-Methylvalerate	0.4 ± 0.1	0.4 ± 0.1	0.5 ± 0.2	0.4 ± 0.1	0.26
4-Methylvalerate	0.2 ± 0.1	0.2 ± 0.1	0.3 ± 0.1	0.3 ± 0.1	0.61
Hexanoate	14.8 ± 2.7	14.7 ± 2.5	19.1 ± 6.0	14.9 ± 4.8	0.13
CRP (mg/L)	4.9 ± 2.5	4.7 ± 4.8	4.7 ± 3.3	4.2 ± 3.2	0.73
Inflammatory Cytokine Score	8.1 ± 2.4	8.2 ± 2.3	8.3 ± 3.1	8.9 ± 2.1	0.67
FABP2 (ng/mL)	3.8 ± 2.9	2.8 ± 1.8	3.5 ± 2.8	3.4 ± 2.0	0.15
LBP (µg/mL)	10.9 ± 3.4	9.5 ± 2.8	10.7 ± 5.5	10.9 ± 3.8	0.34

Data are presented as means ± SD

^a^*P* tested differences in absolute change from baseline, between C and Y groups

### Gut microbiota & SCFA profiling

**Fig. 4:**
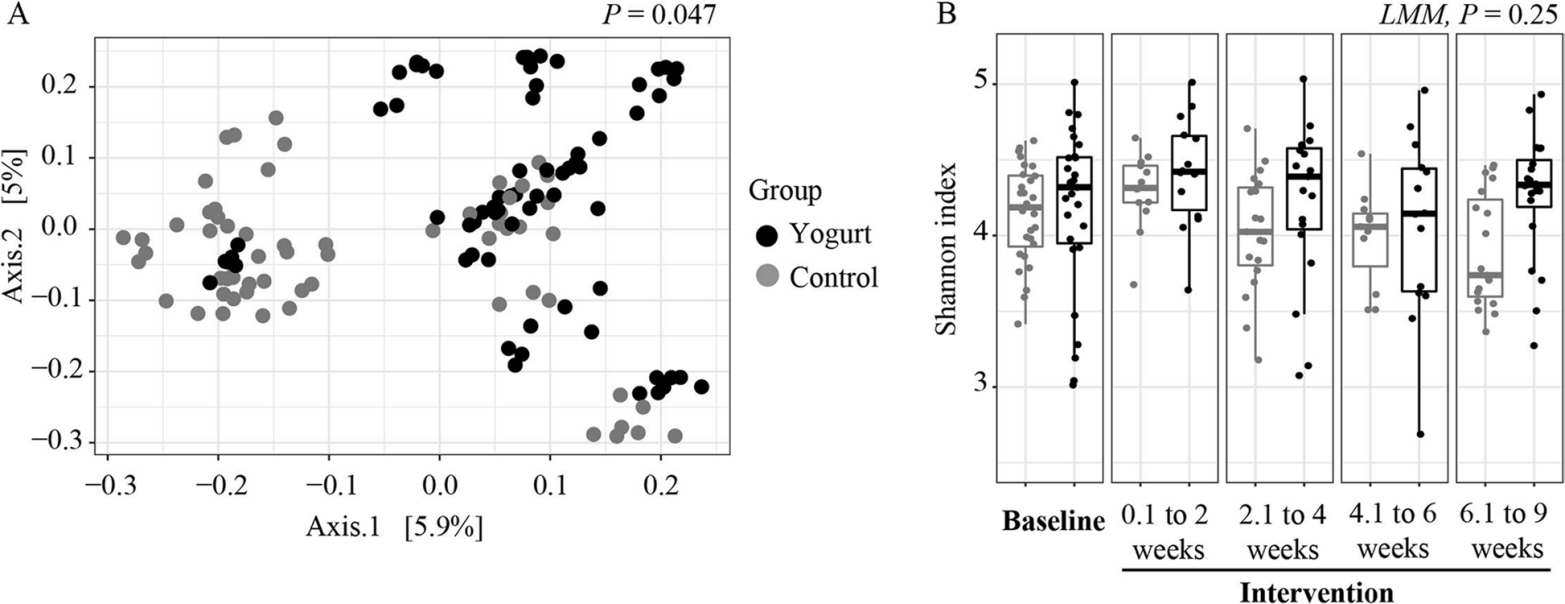
The effect of the daily yogurt supplementation on the gut microbiota among older Caribbean Latino adults compared to a non-yogurt diet-control group. **A** Principal Coordinate Analysis (PCoA) of beta diversity during the intervention showed changes in composition of rare taxa in unweighted UniFrac distance. Analysis was performed comparing control and yogurt groups only within the intervention phase. No baseline samples were included (PERMANOVA, *P* = 0.047). **B** Alpha diversity showed no significant association with daily yogurt consumption when analyzed with linear mixed models only including the yogurt group comparing baseline vs intervention phase (Shannon index, LMM, *P* ≥ 0.25). Although significant differences were observed at 6.1 to 9 weeks (Kruskal–Wallis, *P* = 0.01). Panels **A** and **B** include per subject repeated measures

Across all participants, the baseline microbiota profile was dominated by Firmicutes and Bacteroidetes. Yogurt intake influenced the stool microbial composition compared to the control group when evaluated by unweighted UniFrac distance (*P* = 0.048, R^2^ = 3.5%, PERMANOVA), accounting for repeated measures (as shown in Fig. 4A). However, Bray–Curtis dissimilarity and weighted Unifrac distances were not significant (*P* > 0.05). A total of 25 taxa differed in abundance between yogurt and control groups (*P* ≤ 0.05, MaAsLin, Supplemental Table 5). However, the false discovery rate (FDR) correction for multiple comparisons eliminated significance of all taxa (*P* ≥ 0.28, MaAsLin). Within the yogurt group, 23 differential taxa changed from baseline to intervention, where, as expected, *S. thermophilus* (one of the species present in the yogurt) was higher during the intervention along with other taxa (*P* = 5.6e^−3^, MaAsLin). However, after p-value correction for multiple comparisons all taxa significance was eliminated (*Padj* ≥ 0.42, MaAsLin, Supplemental Table 6).

Yogurt intake did not significantly change the stool microbial alpha diversity (Shannon index) compared to the control group (*P* = 0.25, LMM, Fig. 4B). Additionally, within-group change from baseline to week 8 of intervention was evaluated for both groups, with no significant differences (for beta or alpha diversity,* P* > 0.05, PERMANOVA or LMM respectively). No taxa were associated with absolute change BTMs, inflammatory, and intestinal integrity biomarkers (data not shown). Other variables included in the model, such as sex and BMI showed significance for beta diversity analyses (unweighted Unifrac distance, *P* = 0.01 and *P* = 0.04 respectively, PERMANOVA), however, with a lower R^2^ than for yogurt consumption (R^2^ < 2.7%). No other variables showed significance for alpha diversity (*P* > 0.05, LMM). Additionally, no changes in stool SCFAs were observed, as shown in Table 2.

### Inflammatory & intestinal integrity biomarker assessment

Data for inflammation, evaluated by CRP and inflammatory cytokine score, and intestinal integrity, evaluated by measuring FABP2 and LBP, are presented in Table 2. Overall, the yogurt intervention did not influence these markers.

## Discussion

Yogurt consumption (5-oz daily, containing only *S. thermophilus* and *L. bulgaricus*) for 8 weeks in older Caribbean Latino adults without previous history of yogurt consumption did not improve bone turnover in this population. Yogurt intake did result in rare compositional changes within the gut microbiota; however, alpha diversity and production of SCFAs were not influenced. Finally, markers of inflammation and intestinal integrity were not altered.

Various studies have demonstrated that individuals of Caribbean Latino descent are at high risk for OP and related complications [10, 56, 57]. High concentration of BTMs, including P1NP and OC, may predict bone loss and increased risk of fracture, although these values can be highly variable [58]. Although persons in the current study self-reported no OP at baseline, the mean baseline TRAP5b concentration observed in the current sample of adults is consistent with postmenopausal populations with OP [59]. In a randomized controlled trial (RCT) of 59 post-menopausal women from Iran with type 2 diabetes, low-fat, vitamin D fortified yogurt (2,000 IU per 100 g) supplementation for 12 weeks significantly decreased parathyroid hormone (PTH) and bone resorption marker N-terminal type-1 collagen (NTX), compared to a non-fortified yogurt group [60]. Using a yogurt fortified with vitamin D may explain the observed impact on NTX, versus the lack of impact in the current study on bone markers using a non-fortified yogurt. Further, a RCT in older, White non-Hispanic women demonstrated lower NTX levels following 1-2wk supplementation of 3 servings of yogurt daily [61]. Mean consumption of dairy at baseline in the current sample of adults was 1 serving per day. Provision of the intervention increased total dairy intake to 2 servings per day in the yogurt group. As no significant changes in BTMs were observed with this amount of dairy intake, it is possible that meeting the Dietary Guidelines for Americans’ recommendations [62] for dairy intake at 3 servings per day is necessary, in addition to vitamin D fortification, to cause change in BTMs.

As expected for human stool samples, the baseline microbiota was dominated by Firmicutes and Bacteroidetes [63]. No alpha diversity differences were detected with yogurt consumption, consistent with previous reports [19], likely due to low culture abundances in this yogurt, compared to yogurt enriched with added probiotics beyond *S. thermophilus* and *L. bulgaricus*. The taxon *S. thermophilus*, which was provided in the yogurt, was found higher in the gut of the yogurt intake group; however, analysis of taxa abundance at species level did not yield significant changes between groups or compared to baseline. The composition of the gut microbiota in the yogurt group, compared to the control group, was significantly different when evaluated using unweighted distances. This suggests yogurt influenced the stool microbial composition, most likely of low abundant taxa. These rare taxa may have small or undetected effects on physiological biomarkers measured in this study. However, the lack of significant difference from baseline to week 8 suggests the low or null influence of the yogurt on the gut composition at 141 g per day. Consistent with other studies, the yogurt intervention did not influence diversity and only minor changes to composition were observed between groups [64], leaving the benefits of yogurt consumption on the gut microbiota unclear.

The findings of this study must be interpreted considering some limitations. The study participants were not blinded; however, given the type of intervention study, this would not have been possible. Dietary recalls were performed once weekly and estimated usual intake was assessed from the average of four recalls per month. This is different from standard protocol of assessing diet through two weekdays and one weekend day, which could influence estimated dietary intakes. Clinical markers of bone health, such as PTH and vitamin D, were not measured and limit interpretation of the results. The effect of the intervention on these markers cannot be ruled out and supports the need for a larger sample size and potentially longer duration in a future study. Additionally, due to the vast microbial diversity of a stool sample, non-specific binding of closely related species may have contributed to the relative abundance of *S. thermophilus* and *L. bulgaricus* observed in the qPCR analyses. However, these results are consistent with the increased abundance of *S. thermophilus* in the *16S rRNA* sequencing data, as well as self-reported yogurt consumption, indicating compliance. To optimize participant retention, non-fasted blood was collected, yet the collection of fasted blood in future studies would offer greater flexibility in selecting analytes of interest. Lastly, all medical diagnoses were self-reported.

This study also had several strengths. These include high-quality, and room temperature stability of stool samples for up to 6 weeks post-collection using the OmniGENE kit for microbial DNA preservation for microbiota profiling, measurement of *S. thermophilus* and *L. bulgaricus* to evaluate intervention compliance, measurement of SCFAs to evaluate microbial functionality, and minimal reported yogurt consumption at baseline. In addition to determining a culturally acceptable yogurt prior to study initiation, the selected yogurt met the desired serving size, thus minimizing weight gain. Additionally, diet was routinely monitored throughout the study using a validated assessment method. This provided strength and efficacy when evaluating the effect of the yogurt, independent of normal dietary patterns. Moreover, extensive questionnaires were collected to account for numerous potential covariates. The randomized controlled design eliminated cofounding and selection bias, and paired with routine sampling, allowed for strengthened evaluation of the intervention effect between groups.

## Conclusions

In summary, daily intake of 5-oz of whole fat, yogurt, containing only *S. thermophilus* and *L. bulgaricus*, for 8 weeks did not improve bone turnover in Caribbean Latino men and women. Although, yogurt supplementation was associated with significant differences in beta diversity of the gut microbiota between groups, yogurt supplementation was not associated with significant change of specific taxa, metabolism of SCFA, markers of intestinal integrity nor inflammation. Although yogurt did not influence bone health short-term in this sample of adults, continued research to identify other strategies to improve bone health in this population is warranted due to their high rates of OP.

### Supplementary Information

**Additional file 1: Supplemental Table 1. **Nutrient composition of Oui yogurt provided to the yogurt group. **Supplemental Table 2.** Primer sequences for qPCR used for evaluation of compliance to yogurt or control group assignment. **Supplemental Table 3. **Number of sequences and amplicon sequence variants (ASVs) for the rarefied microbiota data per sample. **Supplemental Table 4.** Comparison of dietary intake at baseline and end of intervention between diet-control (C) and yogurt (Y) group. **Supplemental Table 5.** A total of 25 taxa differed in abundance between diet-control and yogurt-intervention groups post-intervention. **Supplemental Table 6.** A total of 23 taxa differed in abundance from baseline to end of intervention, within the yogurt group.

**Additional file 2.** Baseline Questionnaire.

## Data Availability

Sequences and associated study metadata have been deposited in NCBI with the Sequence Read Archive [65]: SubmissionID SUB11333017 | BioProject ID PRJNA828174 | https://submit.ncbi.nlm.nih.gov/subs/sra/SUB11333017/overview.

## Abbreviations

BMD: Bone mineral density
BTM: Bone turnover markers
CRP: C-reactive protein
EDC: 1-(3-Dimethylaminopropyl)-3-ethylcarbodiimide hydrochloride
FABP2: Intestinal fatty acid binding protein
FDR: False discovery rate
IFN-γ: Interferon gamma
IL: Interleukin
IS: Internal standard
ITT: Intention-to-treat
LBP: Lipopolysaccharide binding protein
LB: *Lactobacillus bulgaricus*
MCP: Monocyte chemoattractant protein
OC: Osteocalcin
OP: Osteoporosis
P1NP: Pro-collagen I alpha 1
qPCR: Quantitative real-time polymerase chain reaction
SCFA: Short chain fatty acids
ST: *Streptococcus thermophilus*
TNF-α: Tumor necrosis factor alpha
TRAP5b: Tartrate-resistant acid phosphatase subunit 5b
3-NPH: 3-Nitrophenylhydrazine
