# Supplementary material for: Daily yogurt consumption does not affect bone turnover markers in men and postmenopausal women of Caribbean Latino descent: a randomized controlled trial
Source: BMC Nutr. 2024 Jan 11;10:12. doi: 10.1186/s40795-023-00800-2 (PMC10785535; doi:10.1186/s40795-023-00800-2)
Supplement: Supplementary file 1 — Additional file 1: Supplemental Table 1. Nutrient composition of Oui yogurt provided to the yogurt group. Supplemental Table 2. Primer sequences for qPCR used for evaluation of compliance to yogurt or control group assignment. Supplemental Table 3. Number of sequences and amplicon sequence variants (ASVs) for the rarefied microbiota data per sample. Supplemental Table 4. Comparison of dietary intake at baseline and end of intervention between diet-control (C) and yogurt (Y) group. Supplemental Table 5. A total of 25 taxa differed in abundance between diet-control and yogurt-intervention groups post-intervention. Supplemental Table 6. A total of 23 taxa differed in abundance from baseline to end of intervention, within the yogurt group. [file 40795_2023_800_MOESM1_ESM.docx]

**Supplemental Table 1)** Nutrient composition of Oui yogurt provided to the yogurt group

| **Nutrients per serving** | **Serving Size: 141 g** |
| --- | --- |
| Energy (kcal) | 170 |
| Total Fat (g) | 8 |
| Total Carbohydrate (g) | 16 |
| Sugars (g) | 15 |
| Protein (g) | 5 |
| Sodium (mg) | 85 |
| Calcium (%) | 15 |
| Vitamin A (%) | 4 |

Ingredients: Pasteurized Grade A Milk, Cane Sugar, Yogurt Cultures (*S. thermophilus*, *L. bulgaricus*), Vanilla Extract.

**Supplemental Table 2)** Primer sequences for qPCR used for evaluation of compliance to yogurt or control group assignment

| **Target** | **Direction** | **Sequence (5’ 🡪 3’)** | **Length** | **Tm (ºC)** |
| --- | --- | --- | --- | --- |
| *S. thermophilus* | Forward | GCTTGTGTTCTGAGGGAAGC | 20 | 56.3 |
| *S. thermophilus* | Reverse | CTTTCTTCTGCACCGTATCCA | 21 | 54.5 |
| *L. bulgaricus* | Forward | GGAAGACTCCGTTTTGGTCA | 20 | 54.9 |
| *L. bulgaricus* | Reverse | AGTTCAAGTCTGCCCCATTG | 20 | 54.0 |
| *16S rRNA* | Forward | AGAGTTTGATCCTGGCTCAG | 20 | 55.2 |
| *16S rRNA* | Reverse | ACGGCTACCTTGTTACGACTT | 21 | 57.4 |

**Supplemental Table 3)** Number of sequences and amplicon sequence variants (ASVs) for the rarefied microbiota data per sample

| **Parameter** | **Count** |
| --- | --- |
| N samples | 184 |
| Total number of seqs | 1,032,608 |
| Mean seqs/sample | 5,612 |
| Standard deviation of seqs/sample | 0 |
| Max number of seqs in a sample | 5,612 |
| Min number of seqs in a sample | 5,612 |
| Total number of ASVs | 6,823 |
| Mean ASV/sample | 271 |
| Standard deviation of ASV/sample | 59 |
| Max number of ASVs in a sample | 422 |
| Min number of ASVs in a sample | 122 |

**Supplemental Table 4**) Comparison of dietary intake at baseline and end of intervention between diet-control (C) and yogurt (Y) group

|  | **Month 0** | | | | | | | **Month 2** | | | | | | |
| --- | --- | --- | --- | --- | --- | --- | --- | --- | --- | --- | --- | --- | --- | --- |
| **Dietary Intake** | **C (*n* = 10)** | | | **Y (*n* = 10)** | | | ***P* ^1^** | **C (*n* = 10)** | | | **Y (*n* = 10)** | | | ***P* ^2^** |
| Total Energy Intake (kcal/d) | 1884 | ± | 432 | 1921 | ± | 348 | 0.84 | 1813 | ± | 472 | 1907 | ± | 672 | 0.72 |
| Total Fat (g/d) | 61 | ± | 21 | 72 | ± | 16 | 0.21 | 57 | ± | 18 | 75 | ± | 35 | 0.16 |
| Total Protein (g/d) | 77 | ± | 27 | 71 | ± | 12 | 0.53 | 86 | ± | 31 | 89 | ± | 34 | 1.00 |
| Total Carbohydrates (g/d) | 257 | ± | 65 | 254 | ± | 70 | 0.92 | 244 | ± | 73 | 216 | ± | 70 | 0.39 |
| Total Fiber (g/day) | 22 | ± | 8 | 23 | ± | 10 | 0.88 | 22 | ± | 8 | 20 | ± | 9 | 0.56 |
| Total Dietary Calcium (mg/d) | 745 | ± | 311 | 730 | ± | 215 | 0.90 | 680 | ± | 311 | 880 | ± | 293 | 0.16 |
| Total Dietary Vitamin D (µg/d) | 5 | ± | 4 | 4 | ± | 2 | 0.35 | 5 | ± | 2 | 6 | ± | 3 | 0.29 |
| Dairy [excluding intervention yogurt] (servings/d) | 1 | ± | 1 | 1 | ± | 1 | 0.58 | 1 | ± | 1 | 1 | ± | 1 | 0.78 |
| Dairy [including intervention yogurt] (servings/d) | 1 | ± | 1 | 1 | ± | 1 | 0.58 | 1 | ± | 1 | 2 | ± | 1 | 0.19 |

Mean dietary intake, calculated by averaging 3 dietary recalls collected in month 2 are presented as mean ± SD. ^1^ P between groups at month 0. ^2^ P between groups at month 2.

**Supplemental Table 5)** A total of 25 taxa differed in abundance between diet-control and yogurt-intervention groups post-intervention

| **Feature** | **Coef ^1^** | **Std Error** | **N** | **N.not.0 ^2^** | ***P*** | ***Q* ^3^** |
| --- | --- | --- | --- | --- | --- | --- |
| *Christensenella massiliensis* | 4.21 | 1.09 | 129 | 82 | 0.00 | 0.28 |
| *Anaerotruncus rubiinfantis* | 1.34 | 0.38 | 129 | 61 | 0.00 | 0.28 |
| *Alistipes senegalensis* | 1.99 | 0.59 | 129 | 57 | 0.00 | 0.28 |
| *Lutispora thermophila* | 1.44 | 0.44 | 129 | 48 | 0.00 | 0.28 |
| *Eubacterium coprostanoligenes* | 2.33 | 0.75 | 129 | 121 | 0.01 | 0.32 |
| *Senegalimassilia anaerobia* | 0.81 | 0.27 | 129 | 37 | 0.01 | 0.35 |
| *Pseudoflavonifractor phocaeensis* | 1.52 | 0.53 | 129 | 82 | 0.01 | 0.38 |
| *Ruminococcus champanellensis* | 2.71 | 0.98 | 129 | 103 | 0.01 | 0.40 |
| *Acetanaerobacterium elongatum* | 3.35 | 1.27 | 129 | 74 | 0.02 | 0.40 |
| *Bacteroides finegoldii* | -1.60 | 0.61 | 129 | 74 | 0.02 | 0.40 |
| *Desulfohalotomaculum halophilum* | 1.22 | 0.47 | 129 | 28 | 0.02 | 0.40 |
| *Slackia isoflavoniconvertens* | 0.75 | 0.30 | 129 | 22 | 0.02 | 0.40 |
| *Flintibacter butyricus* | 1.33 | 0.53 | 129 | 129 | 0.02 | 0.40 |
| *Paludicola psychrotolerans* | 1.14 | 0.46 | 129 | 60 | 0.02 | 0.40 |
| *Oscillibacter ruminantium* | 1.36 | 0.55 | 129 | 128 | 0.02 | 0.40 |
| *Ihubacter massiliensis* | 1.37 | 0.56 | 129 | 97 | 0.02 | 0.40 |
| *Paraprevotella clara* | 2.71 | 1.15 | 129 | 86 | 0.03 | 0.44 |
| *Erysipelothrix larvae* | -1.91 | 0.81 | 129 | 36 | 0.03 | 0.44 |
| *Parolsenella massiliensis* | 1.03 | 0.45 | 129 | 21 | 0.03 | 0.46 |
| *Howardella ureilytica* | 0.99 | 0.45 | 129 | 35 | 0.04 | 0.51 |
| *Blautia faecicola* | 1.36 | 0.62 | 129 | 110 | 0.04 | 0.51 |
| *Harryflintia acetispora* | 0.58 | 0.27 | 129 | 25 | 0.04 | 0.52 |
| *Mogibacterium neglectum* | 1.37 | 0.64 | 129 | 23 | 0.05 | 0.52 |
| *Lachnobacterium bovis* | 0.92 | 0.43 | 129 | 41 | 0.05 | 0.52 |
| *Anaerobutyricum hallii* | 0.87 | 0.41 | 129 | 124 | 0.05 | 0.52 |

^1^ Model effect size. ^2^ Number of samples with at least one sequence for a particular taxon. ^3^ FDR correction for multiple comparisons. Analyzed with MaAsLin2.

**Supplemental Table 6)** A total of 23 taxa differed in abundance from baseline to end of intervention, within the yogurt group

| **Feature** | **Coef ^1^** | **Std Error** | **N** | **N.not.0 ^2^** | ***P*** | ***Q* ^3^** |
| --- | --- | --- | --- | --- | --- | --- |
| *Duncaniella dubosii* | 0.69 | 0.23 | 94 | 36 | 0.00 | 0.42 |
| *Longibaculum muris* | 0.42 | 0.14 | 94 | 24 | 0.00 | 0.42 |
| ***Streptococcus thermophilus*** | 0.81 | 0.28 | 94 | 30 | 0.01 | 0.42 |
| *Fournierella massiliensis* | -0.34 | 0.13 | 94 | 12 | 0.01 | 0.42 |
| *Rhodospirillum rubrum* | -0.96 | 0.35 | 94 | 19 | 0.01 | 0.42 |
| *Blautia luti* | 0.82 | 0.30 | 94 | 86 | 0.01 | 0.42 |
| *Anaeromassilibacillus senegalensis* | 0.71 | 0.28 | 94 | 57 | 0.01 | 0.49 |
| *Muribaculum intestinale* | 0.44 | 0.17 | 94 | 10 | 0.01 | 0.49 |
| *Ihubacter massiliensis* | 0.85 | 0.34 | 94 | 80 | 0.02 | 0.49 |
| *Faecalibacterium prausnitzii* | 0.57 | 0.23 | 94 | 94 | 0.02 | 0.49 |
| *Oscillibacter valericigenes* | 0.87 | 0.36 | 94 | 93 | 0.02 | 0.50 |
| *Butyrivibrio crossotus* | -0.52 | 0.22 | 94 | 39 | 0.02 | 0.50 |
| *Olsenella uli* | -0.29 | 0.13 | 94 | 10 | 0.02 | 0.52 |
| *Eubacterium coprostanoligenes* | 0.63 | 0.28 | 94 | 93 | 0.03 | 0.56 |
| *Megasphaera elsdenii* | -0.22 | 0.10 | 94 | 14 | 0.03 | 0.56 |
| *Streptococcus lactarius* | -0.20 | 0.09 | 94 | 10 | 0.03 | 0.61 |
| *Prevotella disiens* | -0.41 | 0.19 | 94 | 11 | 0.03 | 0.61 |
| *Mixta theicola* | 0.36 | 0.17 | 94 | 10 | 0.04 | 0.63 |
| *Ruminococcus flavefaciens* | 0.29 | 0.14 | 94 | 22 | 0.04 | 0.63 |
| *Parasutterella excrementihominis* | -0.45 | 0.22 | 94 | 30 | 0.04 | 0.63 |
| *Kineothrix alysoides* | 0.38 | 0.18 | 94 | 94 | 0.04 | 0.63 |
| *Christensenella timonensis* | 0.30 | 0.15 | 94 | 12 | 0.05 | 0.63 |
| *Bacteroides faecichinchillae* | 0.24 | 0.12 | 94 | 16 | 0.05 | 0.63 |

^1^ Model effect size. ^2^ Number of samples with at least one sequence for a particular taxon. ^3^ FDR correction for multiple comparisons. Analyzed with MaAsLin2.
