# Supplementary material for: Daily yogurt consumption does not affect bone turnover markers in men and postmenopausal women of Caribbean Latino descent: a randomized controlled trial
Source: BMC Nutr. 2024 Jan 11;10:12. doi: 10.1186/s40795-023-00800-2 (PMC10785535; doi:10.1186/s40795-023-00800-2)
Supplement: Supplementary file 2 — Additional file 2. Baseline Questionnaire. [file 40795_2023_800_MOESM2_ESM.pdf]

MEDICAL DIAGNOSES

Instructions: *In the following section, I will ask you a series of questions regarding a variety of medical diagnoses.*

**Has a DOCTOR ever told you that you had any of the following illnesses or conditions?**	**NO**	**YES**	**Taking medication for this?**	**Is this condition bothering you currently?**
1. Diabetes?	0	1	0. No 1. Yes	0. No 1. Yes
2. High blood pressure/Hypertension?	0	1	0. No 1. Yes	0. No 1. Yes
3. Overweight/obesity?	0	1	0. No 1. Yes	0. No 1. Yes
4. Arthritis?	0	1	0. No 1. Yes	0. No 1. Yes
5. Osteoporosis (hip fracture)?	0	1	0. No 1. Yes	0. No 1. Yes
6. Heart Attack?	0	1	0. No 1. Yes	0. No 1. Yes
7. Heart Disease (other than heart attack)?	0	1	0. No 1. Yes	0. No 1. Yes
8. Stroke?	0	1	0. No 1. Yes	0. No 1. Yes
9. Respiratory disease (such as emphysema, chronic bronchitis, asthma?)	0	1	0. No 1. Yes	0. No 1. Yes
10. Liver or gallbladder disease?	0	1	0. No 1. Yes	0. No 1. Yes
11. Kidney disease?	0	1	0. No 1. Yes	0. No 1. Yes
12. Stomach/ Intestinal Disorder, Stomach Ulcer (bowel elimination problems)?	0	1	0. No 1. Yes	0. No 1. Yes
13. Parkinson's Disease?	0	1	0. No 1. Yes	0. No 1. Yes
14. Skin Cancer?	0	1	0. No 1. Yes	0. No 1. Yes
15. Other type of Cancer? ___________________	0	1	0. No 1. Yes	0. No 1. Yes
16. Eye Disease: Cataract or Glaucoma?	0	1	0. No 1. Yes	0. No 1. Yes
17. Anxiety?	0	1	0. No 1. Yes	0. No 1. Yes
18. Depression?	0	1	0. No 1. Yes	0. No 1. Yes
19. Seizures, Convulsions?	0	1	0. No 1. Yes	0. No 1. Yes
20. Tuberculosis?	0	1	0. No 1. Yes	0. No 1, Yes
21. Hepatitis (Type A, B, or C)?	0	1	0. No 1. Yes	0. No 1. Yes
22. AIDS/HIV positive?	0	1	0. No 1. Yes	0. No 1. Yes
23. Other ________________________________	0	1	0. No 1. Yes	0. No 1. Yes
24. Other ________________________________	0	1	0. No 1. Yes	0. No 1. Yes

MENOPAUSE (*FEMALES ONLY)*

1. Have you already gone through or are you currently going though menopause?	1. Yes 0. No ***(If NO, go to NEXT SECTION)***
2. **If Subject had a hysterectomy, record the year when- or age at which the Subject underwent the procedure:**	Age _______ Year ________ ***(Go to NEXT SECTION)***
3. How old were you when you had your last menstrual period?	Age________ Year ________

Study ID # __________

PRESCRIPTION MEDICATIONS

INTERVIEWER: *List all prescription medications the Subject is currently taking or has taken within the past year, including insulin.*

**Medication Name**	**How long using?**<br>***Codes:*** 1. Less than 1 year (<1yr)<br>2. Between 1 and 5 years (1-5yrs)<br>3. More than 5 years (>5yrs)
1.	1. <1 yr 2. 1-5 yrs 3. >5 yrs
2.	1. <1 yr 2. 1-5 yrs 3. >5 yrs
3.	1. <1 yr 2. 1-5 yrs 3. >5 yrs
4.	1. <1 yr 2. 1-5 yrs 3. >5 yrs
5.	1. <1 yr 2. 1-5 yrs 3. >5 yrs
6.	1. <1 yr 2. 1-5 yrs 3. >5 yrs
7.	1. <1 yr 2. 1-5 yrs 3. >5 yrs
8.	1. <1 yr 2. 1-5 yrs 3. >5 yrs
9.	1. <1 yr 2. 1-5 yrs 3. >5 yrs
10.	1. <1 yr 2. 1-5 yrs 3. >5 yrs
11.	1. <1 yr 2. 1-5 yrs 3. >5 yrs
12.	1. <1 yr 2. 1-5 yrs 3. >5 yrs
13.	1. <1 yr 2. 1-5 yrs 3. >5 yrs
14.	1. <1 yr 2. 1-5 yrs 3. >5 yrs
15.	1. <1 yr 2. 1-5 yrs 3. >5 yrs
16.	1. <1 yr 2. 1-5 yrs 3. >5 yrs
17.	1. <1 yr 2. 1-5 yrs 3. >5 yrs
18.	1. <1 yr 2. 1-5 yrs 3. >5 yrs
19.	1. <1 yr 2. 1-5 yrs 3. >5 yrs
20.	1. <1 yr 2. 1-5 yrs 3. >5 yrs
21.	1. <1 yr 2. 1-5 yrs 3. >5 yrs
22.	1. <1 yr 2. 1-5 yrs 3. >5 yrs
23.	1. <1 yr 2. 1-5 yrs 3. >5 yrs
24.	1. <1 yr 2. 1-5 yrs 3. >5 yrs
25.	1. <1 yr 2. 1-5 yrs 3. >5 yrs

Study ID # __________

OVER-THE-COUNTER MEDICATIONS

INTERVIEWER: *List all over-the-counter medications the Subject takes on a weekly basis. If possible ask participant to bring all over-the-counter medications to the follow-up visit, <u>including laxatives</u>.*

**Medication Name**
1.
2.
3.
4.
5.
6.
7.
8.
9.
10.
11.
12.
13.
14.
15.
16.
17.
18.
19.
20.
21.
22.
23.
24.
25.

Study ID # __________

HEALTH BEHAVIORS: TOBACCO USE

Instructions: *Now, I would like to ask you about the use of tobacco.*

1. Have you smoked at least a hundred or more cigarettes in your lifetime?	0. No ***(If NO, go to NEXT SECTION)*** 1. Yes
2. How old were you when you first started smoking?	Age ________ Year ________
3. Do you currently smoke?	0. No ***(If NO, GO TO #5)*** 1. Yes
4. How many cigarettes, cigars, or pipes do you smoke regularly during one day? ***(pack=20 cigarettes)***	1. Cigarettes _____ 2. Cigars ____ 3. Pipes ____ ***(Answer and go to NEXT SECTION)***
5. On average how many cigarettes, cigars, or pipes did you regularly smoke a day? ***(pack=20 cigarettes)***	1. Cigarettes _____ 2. Cigars ____ 3. Pipes ____
6. How old were you when you last smoked or in what year did you stop smoking? ***(If S stopped smoking in the last year, record current age of S)***	Age ______ Year ______

HEALTH BEHAVIORS: ALCOHOL USE

Instructions: *The following questions refer to alcohol consumption, including wine, spirits, liquors like whiskey, gin, rum or vodka, cocktails, and mixed alcoholic beverages.*

1. Have you had at least 12 drinks of any kind of alcohol during your life? *(Do not count small tastes.)*	0. No ***(If NO, go to NEXT SECTION)*** 1. Yes
2. At what age did you begin drinking?	_______ years
3. Presently, do you drink alcohol?	0. No ***(If NO, GO TO #7)*** 1. Yes
**IF CURRENTLY DRINKING:**	
4. On average, how often do you drink <u>any</u> type of alcohol?	A. ___ ___ ___ # days per: B. 1. Week 2. Month 3. Year
5. What do you usually drink? ***(CIRCLE ALL THAT APPLY)***	1. Beer 2. Rum 3. Wine 4. Gin 5. Whiskey 6. Other _________________ _________________(code)
6. On average, on the days that you drink alcohol, how many drinks do you have a day? By a drink, I mean a 12 oz beer, 4 oz glass of wine, or an ounce of liquor.	_______ drinks ***(Answer and go to NEXT SECTION)***
**IF CURRENTLY NOT DRINKING**	
7. For how many years did you drink alcohol?	________ years
8. What did you usually drink? ***(CIRCLE ALL THAT APPLY)***	1. Beer 2. Rum 3. Wine 4. Gin 5. Whiskey 6. Other ________________ ________________ (code)
9. On average, on the days that you drank alcohol, how many drinks did you have a day? By a drink, I mean a 12 oz beer, a 4 oz glass of wine, or an ounce of liquor.	_______ drinks **(Enter 998 if dk)**

EDUCATION AND MARITAL STATUS

1. What is the highest grade you completed in school?	1. No schooling<br>2. Kindergarten to 4th. grade<br>3. 5th. to 6th. grade<br>4. 7th. to 8th. grade<br>5. 9th. grade<br>6. 10th. grade<br>7. 11th. grade<br>8. 12th. Grade<br>9. High school graduate; HS diploma or equivalent/GED<br>10. Some college credit, but less that 1 year<br>11. 1 or more years of college; no degree<br>12. Associate degree; i.e. AA, AS<br>13. Bachelor's degree, i.e. BA, BS, AB<br>14. Masters (i.e. MS, MA, MEng, MBA)<br>15. Professional degree, (i.e. MD, JD, DDS)<br>16. Doctorate degree, (i.e. PhD, EdD)<br>96. Refused<br>97. Don't remember (dr)<br>98. Don't know (dk)
2. **CURRENT MARITAL STATUS:** Which of the following categories best describes your current marital status?<br>**READ ALL CATEGORIES:**<br>1. Married/living as married/spouse in HH<br>2. Spouse not in HH<br>3. Divorced/separated<br>4. Widowed<br>Never married	1. Married/ living as married, spouse in HH<br>2. Married, spouse not in HH<br>3. Divorced/ separated<br>4. Widowed<br>5. Never married

Study ID # __________

HOUSEHOLD INCOME

***Instructions**: I would like for you to tell me about your household income: who contributes to the necessary expenses, and in what way, and how often does each contributor help out? You have no obligation to share this information with me, but remember that all of the information you share with me will be kept completely confidential.*

**Relationship to Subject**	**Source of Income**<br>***CIRCLE ALL THAT APPLY***	**NO**	**YES**	**Amount**<br>***CODES:***<br>***-997 Refused***<br>***-998 dk***	**Frequency**<br>***CODES:***<br>***1. Weekly***<br>***2. Bi-weekly***<br>***3. Monthly***<br>***4. Yearly***
	0. Employment	0	1	$_______.00	________
	1. TANF	0	1	$_______.00	________
	2. SSI	0	1	$_______.00	________
1. Subject/Self	3. SSDI	0	1	$_______.00	________
	4. Child Support	0	1	$_______.00	________
	5. Pension	0	1	$_______.00	________
	6. Retirement	0	1	$_______.00	________
	7. Food Stamps (SNAP)	0	1	$_______.00	________
	8. Other _____________	0	1	$_______.00	________
	9. Other _____________	0	1	$_______.00	________
	0. Employment	0	1	$_______.00	________
	1. TANF	0	1	$_______.00	________
	2. SSI	0	1	$_______.00	________
2. ________	3. SSDI	0	1	$_______.00	________
	4. Child Support	0	1	$_______.00	________
	5. Pension	0	1	$_______.00	________
	6. Retirement	0	1	$_______.00	________
	7. Food Stamps (SNAP)	0	1	$_______.00	________
	8. Other _____________	0	1	$_______.00	________
	9. Other _____________	0	1	$_______.00	________
	0. Employment	0	1	$_______.00	________
	1. TANF	0	1	$_______.00	________
	2. SSI	0	1	$_______.00	________
3. ________	3. SSDI	0	1	$_______.00	________
	4. Child Support	0	1	$_______.00	________
	5. Pension	0	1	$_______.00	________
	6. Retirement	0	1	$_______.00	________
	7. Food Stamps (SNAP)	0	1	$_______.00	________
	8. Other _____________	0	1	$_______.00	________
	9. Other _____________	0	1	$_______.00	________
	0. Employment	0	1	$_______.00	________
	1. TANF	0	1	$_______.00	________
	2. SSI	0	1	$_______.00	________
4. ________	3. SSDI	0	1	$_______.00	________
	4. Child Support	0	1	$_______.00	________
	5. Pension	0	1	$_______.00	________
	6. Retirement	0	1	$_______.00	________
	7. Food Stamps (SNAP)	0	1	$_______.00	________
	8. Other _____________	0	1	$_______.00	________
	9. Other _____________	0	1	$_______.00	________

MIGRATION HISTORY

Study ID # __________

Instructions: *Now I would like to ask you a series of questions regarding your country of origin and other regions you have previously lived in.*

1. Where were your parents born?	A. Father<br>1. Dominican Republic<br>2. Puerto Rico<br>3. United States<br>4. Other:__________<br>97. Don't remember (dr)<br>98. Don't know (dk)<br><br>B. Mother<br>1. Dominican Republic<br>2. Puerto Rico<br>3. United States<br>4. Other: ____________<br>97. Don't remember (dr)<br>98. Don't know (dk)
2. Where were you born?	1. Dominican Republic<br>2. Puerto Rico<br>3. Massachusetts<br>4. New York<br>5. New Jersey<br>6. Other: __________________
3. In what type of surroundings did you spend most of your time growing up?<br>**READ ALL CATEGORIES**	1. Town or city<br>2. Urban area, outside the town or city<br>3. Rural area, outside the city<br>4. Countryside
4. **FROM 2: If S was NOT born in the US:** In what year did you first move to the United States?	A. Year: ___ ___ ___ ___ **(Enter 9998 if dk)**<br>**OR PROMPT FOR AGE**<br>B. Age: ___ ____ ___ years (**Enter 998 if dk)**
5. **FROM 2: If S was NOT born in MA:** In what year did you first move to Massachusetts?<br><br>**If S was born in MA, STOP HERE.**	A. Year: ___ ___ ___ ___ **(Enter 9998 if dk)**<br>**OR PROMPT FOR AGE**<br>B. Age: ___ ____ ___ years (**Enter 998 if dk)**

6. Where were you living just prior to moving to Massachusetts?	1. Dominican Republic<br>2. Puerto Rico<br>3. Other state (if in U.S.)________________________<br>4. Other country______________________________<br>97. Don't remember (dr)<br>98. Don't know (dk)
7. Since leaving__________ *(country/state of birth)* have you ever gone back to live there for more than three months at a time?	1. Yes 0. No ***(If NO, GO TO #10)***
8. How many times have you gone back to live in ____________ (*country/state of birth*) for more than three months?	________ times **(Enter 998 if dk)**
9. Do you expect to move back to ________________ (*country/state of birth*) in the future?	1. Yes 0. No ***(If NO, GO TO #12)***
10. **If YES:** When/in what year?	____ ____ ____ ____ year **(Enter 9998 if dk)**

Study ID # __________

ANTHROPOMETRY SECTION

Instructions: *Now I will take measurements of your weight and height.*

1. Have you lost or gained weight in the last 6 months?	1. Yes 0. No ***(If NO, GO TO #5)*** 98. Don't know (dk)
2. **If YES:** How many pounds have you lost or gained?	**a)** __________ lbs. **(Enter 998 if dk)**<br>b) 1. Lost<br>2. Gained
3. Was the weight loss/gain intentional?	1. Yes 0. No 98. Don't know (dk)<br>***(If YES, GO TO #5)***
4. **If NO:** Why do you think you lost or gained weight?	______________________________<br>______________________________
5. Weight:<br>997 - not performed for safety reasons<br>998 - subject refused measurement<br>999 - unable to obtain measurement	a) ___________.______lbs.<br>b) ___________.______lbs.
6. Standing Height:<br>997 - not performed for safety reasons<br>998 - subject refused measurement<br>999 - unable to obtain measurement	a) ___________.______cm.<br>b) ___________.______cm.

Study ID # __________

ACCULTURATION

Instructions: *The following questions have the purpose of exploring acculturation and assimilation to this country.*

1. Which is your native language?	1. English<br>2. Spanish<br>3. Other<br>Specify: ______________________________
2. What languages do you speak?	a. English 1. Yes 2. No 3. A little<br>b. Spanish 1. Yes 2. No 3. A little<br>c. Other 1. Yes 2. No 3. A little<br>Specify: ______________________________
3. Would you say that you use mostly English or mostly Spanish or both about the same?	1. Only English<br>2. Only Spanish<br>3. Mostly English<br>4. Mostly Spanish<br>5. Both the same
4. **If YES or PARTIALLY to both previous questions:** Which language do you write better?	1. English<br>2. Spanish<br>3. Both equally

5. What language do you use:		Only Spanish	More Spanish than English	Both equally	More English than Spanish	Only English	NA
5A...for watching TV?	A.	1	2	3	4	5	6
5B...for reading newspapers/books?	B.	1	2	3	4	5	6
5C...for speaking with neighbors?	C.	1	2	3	4	5	6
5D...at work?	D.	1	2	3	4	5	6
5E...for listening to the radio?	E.	1	2	3	4	5	6
5F...with friends?	F.	1	2	3	4	5	6
5G...with family?	G.	1	2	3	4	5	6

Study ID # __________

BOWEL HEALTH

Instructions: *Next, we'd like to talk to you about your bowel health. First we will start by asking how often you have a bowel movement.*

1. How often do you usually have bowel movements?<br><br>PROBE: How many times per day or per week do you usually have a bowel movement?	1a. Enter number of times:<br>1. __________<br>2. Refused<br>3. Don't know<br><br>1b. Enter unit:<br>1. Day<br>2. Week
2. Please tell me what number corresponds to your usual or most common stool type.<br><br>PROBE: Show numeric description to Subject if needed.	1. Type 1 – separate hard lumps, like nuts<br>2. Type 2 – sausage-like, but lumpy<br>3. Type 3 – sausage-like but with cracks in the surface<br>4. Type 4 – sausage-like or snake, smooth and soft<br>5. Type 5 – soft blobs with clear, cut edges<br>6. Type 6 – fluffy pieces with ragged edges, a mushy stool<br>7. Type 7 – watery, no solid pieces<br>8. Refused<br>9. Don't know
3. During the past 12 months how often have you had an urgent need to empty your bowels that makes you rush to the toilet?	1. always<br>2. most of the time<br>3. sometimes<br>4. rarely<br>5. never<br>6. refused<br>7. don't know
4. During the past 12 months how often have you been constipated?<br><br>PROBE: constipation being defined as no bowel movement for 3 or more days	1. always<br>2. most of the time<br>3. sometimes<br>4. rarely<br>5. never<br>6. refused<br>7. don't know
5. During the past 12 months how often have you had diarrhea?<br><br>PROBE: diarrhea being defined as loose, watery and possibly more-frequent bowel movements than usual	1. always<br>2. most of the time<br>3. sometimes<br>4. rarely<br>5. never<br>6. refused<br>7. don't know
6. Have you taken laxatives or stool softeners in the **past 30 days**? Would you say…<br><br>[If yes, move to question 7. If none, stop interview]	1. most days<br>2. 1-3 times a week<br>3. 2-3 times a month<br>4. once per month<br>5. never<br>6. refused<br>7. don't know

[If answered yes to laxative use in question 6]<br>7. What type of laxative did you use in the past 30 days?<br><br>**[If participant has bottle readily available ask to see the bottle.]**	Name laxative [brand]: ___________________
8. What amount of laxative did you take?<br><br>PROBE: If Subject has taken a laxative multiple times in the past 30 days, document the most recent time and amount.	Amount of laxative: ___________________<br><br>Date of last use: ___________________
9. Why did you take a laxative in the past 30 days?	1. Due to constipation<br>2. To ‘cleanse’ the body<br>3. Other: _________________________________

Study ID # __________

(ACT) PHYSICAL ACTIVITY

***Instructions**: Now, I would like to ask you about the different activities you do every day. I will read out loud a list of daily activities, and I would like for you to tell me how many hours, approximately, you spend every day on each given activity. Let's think about this past week as an example.*

**CO-INFORMANT:** YES _____ NO _____		
Last week, on a USUAL WEEKDAY (we will do the same for a WEEKEND DAY afterwards), how much time did you spend…:	***Hours per day for a usual WEEKDAY:***<br>A	***Hours per day for a usual WEEKEND day:***<br>B
1. **SLEEPING AND LYING DOWN** (even if not sleeping: night-time sleep, naps and reclining)<br>**ASK EACH SEPARATELY, THEN SUM.**		
2. **VIGOROUS ACTIVITY:** (brisk walking, digging in the garden, strenuous sports, jogging, sustained swimming, chopping wood, heavy carpentry, bicycling on hills, etc.)		
3. **MODERATE ACTIVITY:** (heavy housework, light sports, regular walking, dancing, yard work, painting, repairing, light carpentry, bicycling on level ground, etc.)		
4. **LIGHT ACTIVITY:** (office work, light housework, driving a car, strolling, personal care, standing with little motion etc.)		
5. **SITTING ACTIVITY:** (eating, reading, watching TV, listening to the radio etc.)		
**REPEAT QUESTIONS ABOVE FOR COLUMN B ANSWERS**		
6. **TOTAL:**<br>(**NOTE**: Total for each day should add up to 24 hours).		

7. Would you say that during the past week you were less active than usual, more active, or about as active as usual?	1. Less active than usual<br>2. More active than usual<br>3. As active as usual
8. How many <u>flights</u> of stairs do you climb up each day?	______ flights
9. How many city blocks or their equivalent do you walk each day?	_______ blocks<br>**OR**<br>_______ minutes
10. How much time do you spend watching TV each day?	_______hours

Anthropometry Section

Record ID ______________________

ANTHROPOMETRY

"Now I will ask you a few questions about changes in weight and take your weight"

Weight: ______________________ ((lbs))

997 - not performed for safety reasons
998 - subject refused measurement
999 - unable to obtain measurement

If you lost or gained weight since your last visit, was it intentional?

- ◯ Yes
- ◯ No
- ◯ Don't know

Why do you think you lost or gained weight? ______________________

Health Behaviors

Record ID ______________________________

TOBACCO USE:

"Now I'll ask you about the use of tobacco"

Have you started smoking since your last visit?
- ◯ Yes
- ◯ No

How many cigarettes, cigars, or pipes do you smoke regularly during one day? (pack=20 cigarettes) ______________________________
(number & specify if cigar, cigrette, or pipe)

Medical Diagnosis

Record ID ______________________

MEDICAL DIAGNOSES

"Since your last visit, has a DOCTOR told you that you have any of the following illnesses or conditions?"

	Yes	No
Diabetes	○	○
High blood pressure/ Hypertension	○	○
Overweight/ Obesity	○	○
Arthritis	○	○
Osteoporosis	○	○
Heart Attack	○	○
Heart Disease (other than heart attack)	○	○
Stroke	○	○
Respiratory disease (such as emphysema, chronic bronchitis, asthma)	○	○
Liver or gallbladder disease	○	○
Kidney disease	○	○
Stomach/ intestinal disorder, stomach ulcer (bowel elimination problems)	○	○
Parkinson's Disease	○	○
Skin cancer	○	○
Other type of cancer	○	○
Eye disease: Cataract or glaucoma	○	○
Anxiety	○	○
Depression	○	○
Siezures, convulsions	○	○
Tuberculosis	○	○
Hepatitis (Type A, B, or C)	○	○
Aids/HIV positive	○	○
Other?	○	○

Taking medication for this?
○ Yes
○ No

Is this condition bothering you currently?	◯ Yes<br>◯ No
Taking medication for this?	◯ Yes<br>◯ No
Is this condition bothering you currently?	◯ Yes<br>◯ No
Is this condition bothering you currently?	◯ Yes<br>◯ No
Taking medication for this?	◯ Yes<br>◯ No
Is this condition bothering you currently?	◯ Yes<br>◯ No
Taking medication for this?	◯ Yes<br>◯ No
Is this condition bothering you currently?	◯ Yes<br>◯ No
Taking medication for this?	◯ Yes<br>◯ No
Is this condition bothering you currently?	◯ Yes<br>◯ No
Taking medication for this?	◯ Yes<br>◯ No
Is this condition bothering you currently?	◯ Yes<br>◯ No
Taking medication for this?	◯ Yes<br>◯ No
Is this condition bothering you currently?	◯ Yes<br>◯ No
Taking medication for this?	◯ Yes<br>◯ No
Is this condition bothering you currently?	◯ Yes<br>◯ No
Taking medication for this?	◯ Yes<br>◯ No
Is this condition bothering you currently?	◯ Yes<br>◯ No

Taking medication for this?	◯ Yes<br>◯ No
Is this condition bothering you currently?	◯ Yes<br>◯ No
Taking medication for this?	◯ Yes<br>◯ No
Is this condition bothering you currently?	◯ Yes<br>◯ No
Taking medication for this?	◯ Yes<br>◯ No
Is this condition bothering you currently?	◯ Yes<br>◯ No
Taking medication for this?	◯ Yes<br>◯ No
Is this condition bothering you currently?	◯ Yes<br>◯ No
What kind of cancer?	__________________________________
Taking medication for this?	◯ Yes<br>◯ No
Is this condition bothering you currently?	◯ Yes<br>◯ No
Taking medication for this?	◯ Yes<br>◯ No
Is this condition bothering you currently?	◯ Yes<br>◯ No
Taking medication for this?	◯ Yes<br>◯ No
Is this condition bothering you currently?	◯ Yes<br>◯ No
Taking medication for this?	◯ Yes<br>◯ No
Is this condition bothering you currently?	◯ Yes<br>◯ No
Taking medication for this?	◯ Yes<br>◯ No

Is this condition bothering you currently?	◯ Yes<br>◯ No
Taking medication for this?	◯ Yes<br>◯ No
Is this condition bothering you currently?	◯ Yes<br>◯ No
Taking medication for this?	◯ Yes<br>◯ No
Is this condition bothering you currently?	◯ Yes<br>◯ No
Taking medication for this?	◯ Yes<br>◯ No
Is this condition bothering you currently?	◯ Yes<br>◯ No
Specify other:	__________________________
Taking medication for this?	◯ Yes<br>◯ No
Is this condition bothering you currently?	◯ Yes<br>◯ No

Medications

Record ID __________________________________

PRESCRIPTION MEDICATIONS

"Has there been any changes to your medication/medications since your last visit? "

Medication name? __________________________________

How long have you been taking this?
- ○ less than one year
- ○ between 1 and 5 years
- ○ more than 5 years

Medication name? __________________________________

How long have you been taking this?
- ○ less than one year
- ○ between 1 and 5 years
- ○ more than 5 years

Medication name? __________________________________

How long have you been taking this?
- ○ less than one year
- ○ between 1 and 5 years
- ○ more than 5 years

Medication name? __________________________________

How long have you been taking this?
- ○ less than one year
- ○ between 1 and 5 years
- ○ more than 5 years

Medication name? __________________________________

How long have you been taking this?
- ○ less than one year
- ○ between 1 and 5 years
- ○ more than 5 years

Medication name? __________________________________

How long have you been taking this?
- ○ less than one year
- ○ between 1 and 5 years
- ○ more than 5 years

Medication name? __________________________________

How long have you been taking this?	○ less than one year<br>○ between 1 and 5 years<br>○ more than 5 years
Medication name?	________________________
How long have you been taking this?	○ less than one year<br>○ between 1 and 5 years<br>○ more than 5 years
Medication name?	________________________
How long have you been taking this?	○ less than one year<br>○ between 1 and 5 years<br>○ more than 5 years
Medication name?	________________________
How long have you been taking this?	○ less than one year<br>○ between 1 and 5 years<br>○ more than 5 years

OVER THE COUNTER MEDICATIONS

"Have you started taking any NEW over-the-counter medications since your last visit? If it's possible I ask that you bring all over-the-counter medications to our follow-up visit, including laxatives."

Medication name?	________________________
Medication name?	________________________
Medication name?	________________________
Medication name?	________________________
Medication name?	________________________
Medication name?	________________________
Medication name?	________________________

What type of laxative did you use in the last week?

[If participant has bottle readily available ask to see the bottle.]

______________________ (Name/brand)

What amount of laxative did you take?

______________________ (dosage or amount taken)

Date of last use:

PROBE: If Subject has taken a laxative multiple times in the past 30 days, document the most recent time and amount.

______________________ (D-M-Y)

Why did you take a laxative in the past week?

- ◯ Due to constipation
- ◯ To 'cleanse' the body
- ◯ Other

Medication name? ________________

Medication name? ________________

Medication name? ________________

Medication name? ________________

Medication name? ________________

Medication name? ________________

Medication name? ________________

Medication name? ________________

DIETARY SUPPLEMENTS

"Now I'll ask you to list any NEW dietary supplements since your last visit"

Supplement name: ________________

Supplement name: ________________

Supplement name: ________________

Supplement name: ________________

Supplement name: ________________

Supplement name: ________________

Supplement name: ________________

Supplement name: ________________

Supplement name:

Supplement name:

Bowel Health

Record ID

____

BOWEL HEALTH

"Now I will ask you about your bowel health since your last visit"

Have their been any changes to your bowel movements since your last visit?

____
(Specify per day or week)

Please tell me what number corresponds to your usual or most common stool type over the past week.

PROBE: Show numeric description to Subject if needed.

- ◯ Type 1 - separate hard lumps, like nuts
- ◯ Type 2 - sausage-like, but lumpy
- ◯ Type 3 - sausage-like but with cracks in the surface
- ◯ Type 4 - sausage-like or snake, smooth and soft
- ◯ Type 5 - soft blobs with clear, cut edges
- ◯ Type 6 - fluffy pieces with ragged edges, a mushy stool
- ◯ Type 7 - watery, no solid pieces
- ◯ Refused
- ◯ Don't know

During the past week how often have you had an urgent need to empty your bowels that makes you rush to the toilet?

- ◯ Always
- ◯ Most of the time
- ◯ Sometimes
- ◯ Rarely
- ◯ Never
- ◯ Refused
- ◯ Don't know

During the past week how often have you been constipated?

PROBE: constipation being defined as no bowel movement for 3 or more days

- ◯ Always
- ◯ Most of the time
- ◯ Sometimes
- ◯ Rarely
- ◯ Never
- ◯ Refused
- ◯ Don't know

During the past week how often have you had diarrhea?

PROBE: diarrhea being defined as loose, watery and possibly more-frequent bowel movements than usual

- ◯ Always
- ◯ Most of the time
- ◯ Sometimes
- ◯ Rarely
- ◯ Never
- ◯ Refused
- ◯ Don't know

Have you taken laxatives or stool softeners in the past week? Would you say...

- ◯ Most days
- ◯ 1-3 times a week
- ◯ 2-3 times a month
- ◯ Once per month
- ◯ Never
- ◯ Refused
- ◯ Don't know
